# RETRACTED: Kim et al. Anthocyanins Derived from *Vitis coignetiae Pulliat* Contributes Anti-Cancer Effects by Suppressing NF-κB Pathways in Hep3B Human Hepatocellular Carcinoma Cells and In Vivo. *Molecules* 2020, *25*, 5445

**DOI:** 10.3390/molecules31203778

**Published:** 2026-10-21

**Authors:** Min Jeong Kim, Anjugam Paramanantham, Won Sup Lee, Jeong Won Yun, Seong Hwan Chang, Dong Chul Kim, Hyeon Soo Park, Yung Hyun Choi, Gon Sup Kim, Chung Ho Ryu, Sung Chul Shin, Soon Chan Hong

**Affiliations:** 1 Departments of Internal Medicine, Institute of Health Sciences, Gyeongsang National University Hospital, Gyeongsang National University School of Medicine, 90 Chilam-dong, Jinju 660-702, Republic of Korea; bokdae@hanmail.net (M.J.K.); anju.udhay@gmail.com (A.P.); potato-yun@hanmail.net (J.W.Y.); 2 School of Veterinary, Research Institute of Life Science, Gyeongsang National University, 501 Jinju-daero, Jinju 52828, Republic of Korea; dion5280@naver.com (H.S.P.); gonskim@gnu.ac.kr (G.S.K.); 3 Department of Surgery, Konkuk University School of Medicine, Seoul 143-701, Republic of Korea; csh@kuh.ac.kr; 4 Departments of Pathology, Institute of Health Sciences, Gyeongsang National University School of Medicine, 90 Chilam-dong, Jinju 660-702, Republic of Korea; kdcjes@hanmail.net; 5 Department of Biochemistry, Dongeui University College of Oriental Medicine, 42 San, Yangjung-dong, Busan 614-052, Republic of Korea; choiyh@deu.ac.kr; 6 Department of Biomaterial Control (BK21 Program), Dongeui University Graduate School, 42 San, Yangjung-dong, Busan 614-052, Republic of Korea; 7 Department of Food Technology, Research Institute of Life Science, Gyeongsang National University, 900 Gajwadong, Jinju 660-701, Republic of Korea; ryu@gsnu.ac.kr; 8 School of Chemistry, Research Institute of Life Science, Gyeongsang National University, 900 Gajwadong, Jinju 660-701, Republic of Korea; scshin@gsnu.ac.kr; 9 Department of Surgery, Institute of Health Sciences, Gyeongsang National University School of Medicine, 90 Chilam-dong, Jinju 660-702, Republic of Korea; hongsc@gnu.ac.kr

The journal retracts the article titled “Anthocyanins Derived from *Vitis coignetiae Pulliat* Contributes Anti-Cancer Effects by Suppressing NF-κB Pathways in Hep3B Human Hepatocellular Carcinoma Cells and In Vivo” [1], cited above.

Following publication, concerns were brought to the attention of the Editorial Office regarding the integrity of a figure presented in this article.

According to the journal’s standard procedures, the Editorial Office and Editorial Board conducted an investigation that identified indications of image duplications and inappropriate editing within Figure 1B, as well as incomplete reporting of the ethical approval information.

Although the authors cooperated during the investigation, the explanations and supporting materials provided were not deemed sufficient by the Editorial Board to resolve the identified concerns. As a result, the Editorial Board has decided to retract this paper as per MDPI’s retraction policy.

The authors requested voluntary retraction after determining that the available original records and documentation were insufficient to fully address the concerns raised. The authors explained that certain original unprocessed image files and individual documents relating to ethical approval could not be retrieved from the institutional archives.

This retraction was approved by the Editor-in-Chief of the journal *Molecules*.

The authors have cooperated in the investigation and agree with the retraction.

## References

[1] Kim M.J., Paramanantham A., Lee W.S., Yun J.W., Chang S.H., Kim D.C., Park H.S., Choi Y.H., Kim G.S., Ryu C.H. (2020). RETRACTED: Anthocyanins Derived from *Vitis coignetiae Pulliat* Contributes Anti-Cancer Effects by Suppressing NF-κB Pathways in Hep3B Human Hepatocellular Carcinoma Cells and In Vivo. Molecules.

